# Probiotic‐Mediated Vitamin D Supplementation Improves the Gut Microbiota and Vitamin D Receptor Composition in Obese Rats

**DOI:** 10.1002/fsn3.71967

**Published:** 2026-06-01

**Authors:** Gül Eda Kılınç, Mehtap Ünlü Söğüt

**Affiliations:** ^1^ Faculty of Health Sciences, Department of Nutrition and Dietetics Ondokuz Mayıs University Samsun Turkey

**Keywords:** gut microbiota, obesity, probiotics, vitamin D, vitamin D receptor

## Abstract

Obesity is a complex disorder affecting metabolism and immunity, with gut microbiota and vitamin D metabolism playing key roles. This study aimed to evaluate the impact of vitamin D supplementation and probiotics on the vitamin D receptor (VDR) levels and the gut microbiota of rats given a high‐fat diet as an experimental model of obesity. In this study, experimental obese rat models were used to assess the impact of vitamin D and probiotic supplements on lipid profiles, inflammation, obesity, diabetes, VDR, and gut microbiota. 24 Wistar rats (4–6 weeks) were divided into four groups: control, high‐fat diet, high‐fat diet with probiotic supplementation (HFD + P), and high‐fat diet with probiotic and vitamin D supplementation (HFD + P + VD). Blood and fecal samples were collected from rats for analysis. Changes in BMI values, initial and final weights, and weight changes across all groups were found (*p* = 0.018, *p* = 0.006, *p* = 0.023). HFD + P and HFD + P + VD showed lower levels of insulin, CRP, triglycerides, IL‐6, HOMA‐IR, and leptin. Supplementing probiotics and vitamin D reduced the phylum *Bacteroidetes* while increasing the phyla *Proteobacteria, Firmicutes,* and *Actinobacteria.* The findings suggest that probiotics combined with vitamin D supplementation may be a promising approach for supporting the management of obesity‐related metabolic disorders and microbiota. Furthermore, probiotics may enhance vitamin D's effectiveness by inducing VDR expression.

## Graphical abstract

Lifestyle factors such as regular physical activity, dietary fiber intake, Mediterranean‐style eating patterns, and breastfeeding support gut microbiota balance, whereas Western‐style diets, fast‐food consumption, sugar‐sweetened beverages, and excessive screen time contribute to dysbiosis and obesity. Obesity‐associated vitamin D deficiency impairs intestinal barrier integrity, increases inflammation and insulin resistance, and disrupts microbial composition. Combined probiotic and vitamin D supplementation may help restore gut microbiota balance by increasing beneficial bacteria, improving intestinal barrier function, reducing circulating lipopolysaccharides and inflammation, and supporting metabolic health.

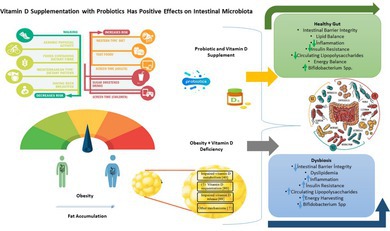

## 1 Introduction

A serious public health issue, obesity causes glucose and lipid metabolism disorders characterized by irregularities in energy homeostasis, paving the way for the development of chronic diseases. Due to its various functions, the gut microbiota is associated with many chronic diseases, including obesity (Geng et al. 2022; Rohm et al. 2022). Despite several different pathways having been proposed to describe the relationship between intestinal microbiota and obesity, which maintains a symbiotic life with humans and provides physiological homeostasis, it is reported that it has functional effects, especially on body weight, and its role in energy homeostasis is remarkable (Berland et al. 2022; Islam et al. 2022). In recent years, the potential roles of biological agents such as probiotics and vitamin D in obesity management have attracted attention. However, this area has some mechanistic uncertainties and inconsistent results (Jürgenson et al. 2025; Kim et al. 2025; Lee 2025; Liu et al. 2025).

In recent years, among the essential subjects studied for preventing obesity‐related diseases or supporting treatment is vitamin D, a fat‐soluble steroid hormone. Vitamin D affects obesity through various mechanisms, and studies show that adipose tissue expansion is triggered in cases of vitamin D deficiency (Szymczak‐Pajor et al. 2022; Zakharova et al. 2019). The vitamin D receptor (VDR) is essential for vitamin D to exert its biological effects and is a receptor expressed by many cells. It has been reported that polymorphisms or mutations in the VDR gene may cause obesity (Alzaim et al. 2022; Faghfouri et al. 2022). The identification of the VDR in many tissues across the body has revealed new insights into vitamin D's functions. The intestinal lumen is among these tissues, and it is suggested that vitamin D may affect the microbiota in various ways (Sun and Zhang 2022; Wang et al. 2022).

When probiotics and vitamin D are considered together, there are uncertainties and limitations regarding their interactions via biological pathways. Probiotics may influence gut microbial activity by enhancing short‐chain fatty acid production, thereby attenuating inflammatory responses. In contrast, vitamin D exerts regulatory effects predominantly through VDR‐mediated signaling pathways that support intestinal barrier integrity and modulate inflammatory cascades, such as NF‐κB. In addition to these distinct mechanisms, evidence suggests that microbiota‐derived metabolites, particularly butyrate, can upregulate VDR expression, indicating a functional interaction between gut microbial activity and vitamin D signaling. Collectively, these mechanistic links provide a biologically plausible rationale for investigating the combined effects of probiotics and vitamin D on obesity‐related metabolic regulation (Li et al. 2025; Parada Venegas et al. 2019; Wu, Weng, et al. 2015). Specifically, butyrate supplementation has reportedly improved colonic inflammation by increasing VDR gene expression and protein production (Wu, Yoon, et al. 2015). This anti‐inflammatory effect of butyrate has been shown to contribute to VDR signaling by activating G‐protein‐coupled receptors (GPCRs) on the cell surface, such as GPR109A, GPR41, and GPR43 (Parada Venegas et al. 2019). The effects of probiotic strains on VDR have been investigated in various studies. *Lactobacillus plantarum* and *Lactobacillus rhamnosus* GG strains have been shown to boost VDR expression and activity in various cell lines. These effects are protective only in mice of the wild type with intact VDR function in development of *Salmonella*‐induced colitis (Wu, Weng, et al. 2015). In a study evaluating the effects of specific probiotic strains, only *L. plantarum* among six strains of *Lactobacillus* were shown to be considerably increased VDR expression in HT‐29 MTX cells. Still, the mechanism by which this increase is reflected in functional activity remains incompletely elucidated (Raveschot et al. 2020). In studies using the TNBS‐induced inflammation‐cancer transition model, it was reported that administering VSL#3 (a multi‐strain probiotic formulation) delayed inflammation‐related dysplasia and carcinogenesis by increasing VDR expression (Appleyard et al. 2011). In addition, identical probiotic blend was demonstrated to reduce insulin resistance by causing different nuclear receptors to be expressed, including VDR, in liver and adipose tissues in animals using a genetic dyslipidemia model and to provide a protective effect against development of atherosclerosis and steatohepatitis (Mencarelli et al. 2012). In a study, administration of *Lactobacillus casei* BL23 was reported to activate homeostasis‐related genes, including VDR‐α, thereby positively affecting development of the immune system, growth, and survival (Qin et al. 2018). On the other hand, some experimental and clinical evidence indicates that probiotics raise vitamin D levels, VDR expression, and activity. For example, in a randomized controlled study examining the potential to reduce cholesterol of *Lactobacillus reuteri* NCIMB 30242, which has bile salt hydrolase activity, post hoc analyses showed that this probiotic significantly increased plasma 25‐hydroxyvitamin D levels 9 weeks following delivery, without negatively impacting fat‐soluble vitamin absorption (Jones et al. 2013). Previous studies in animal models have shown that vitamin D and probiotic supplementation can improve gut microbiota composition, metabolic parameters, and inflammatory status via VDR‐related and gut axis mechanisms. In contrast, clinical studies report more heterogeneous and generally modest effects on metabolic and inflammatory outcomes. These differences are likely due to species‐specific physiology, study design, and baseline metabolic status (Abboud et al. 2020; Franks et al. 2024; Ostadmohammadi et al. 2019; Raygan et al. 2018).

In summary, although the effects of probiotics and vitamin D on metabolic and inflammatory processes are well documented separately, the mechanisms underlying their combined effects on obesity remain poorly elucidated. Considering this information, combining probiotics and vitamin D may exert synergistic effects by enhancing VDR‐mediated signaling pathways, thereby improving obesity‐related metabolic and inflammatory outcomes more effectively than either intervention alone. In this study, we hypothesize that probiotic‐supported vitamin D administration will have a greater impact on obesity‐related inflammation, metabolic disorders, and body composition than probiotic or vitamin D administration alone. In an experimentally produced obesity model in rats fed a high‐fat diet, the current study sought to evaluate how probiotics and vitamin D supplements affect intestinal microbiota and VDR levels.

## 2 Materials and Methods

### 2.1 Animals

**FIGURE 1:**
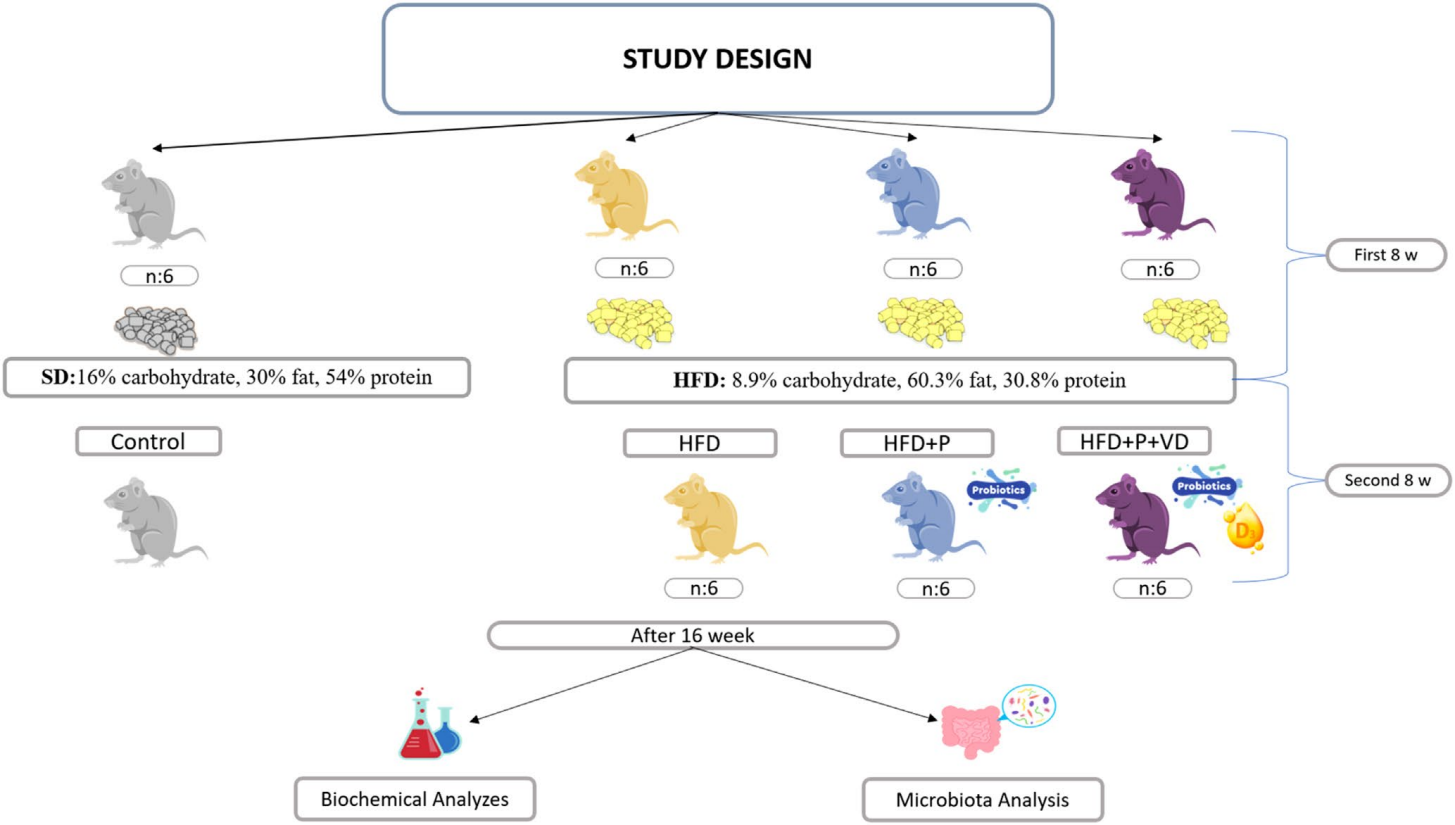
The general study design and dietary components.

Healthy male Wistar rats (4–6 weeks) weighing 270–290 g were purchased from the Experimental Animals Research and Application Center (12 h light and dark cycle, 45%–70% humidity, and 22°C–24°C). Animals were maintained in standard laboratory cages with appropriate bedding under routine husbandry conditions. They had free access to water and food. Before the experiments began, they were allowed to acclimate for 7 days in a controlled environment. Minimal pain and distress for the animals were achieved by careful handling and appropriate anesthesia during the experiments. The animals were sacrificed under deep anesthesia. The ethical approval for this research was obtained from the University Ethics Commission (No: 68489742‐604.01‐E.29664). A summary of the study method is presented in Figure 1. The researchers performed outcome measurements and data analyses; no blinding procedure was applied during the experimental procedures or analysis. All biochemical and anthropometric measurements were performed using standardized laboratory protocols.

### 2.2 Diets

The rats were randomly divided into two groups: the standard diet group (control) (SD, *n* = 6; 16% carbohydrate, 30% fat, and 54% protein; 16 weeks) and the high‐fat diet group (HFD, *n* = 18; 8.9% carbohydrate, 60.3% fat, and 30.8% protein; 8 weeks) (Woods et al. 2003).

### 2.3 Experimental Groups

The experiment lasted 16 weeks and was run in two phases: (1) the creation of the obese rat models (0–8 weeks) and (2) the intervention phase (9–16 weeks). The 24 rats were randomly divided into four subgroups using a computer‐generated random number sequence to ensure unbiased group allocation: control and fed a chow diet (*n* = 6); HFD, fed a high‐fat diet (*n* = 6); HFD + P, fed a high‐fat diet and supplemented with probiotic (*n* = 6); and HFD + P + VD, fed a high‐fat diet and supplemented with probiotics and vitamin D (*n* = 6) for the diet‐induced obesity (DIO) models. The animals were treated daily between 10 a.m. and 11 a.m., and all treatments were performed over eight consecutive weeks. Weight gain (WG), food intake, and body weight were monitored weekly. Body mass index (BMI) was calculated using the formula described by Novelli et al. (2007) and adapted from standard rodent anthropometric assessments. Body weight (g) and nose‐to‐anus length (cm) were measured, and BMI was determined as body weight divided by the square of body length (BMI = g/cm^2^). In addition to BMI, body length was used as a standard morphological parameter for normalization of body mass. BMI values were expressed in g/cm^2^, and a value above 0.68 g/cm^2^ was considered indicative of obesity in rats (Novelli et al. 2007).

### 2.4 Probiotic Supplement and Vitamin D Supplement

The probiotic supplementation consisted of a multi‐strain mixture including *Lactobacillus rhamnosus* GG, *Lactobacillus rhamnosus* LGG, *Lactobacillus acidophilus* LA‐*5, Lactobacillus paracasei* LCASEI431, and *Bifidobacterium lactis* BB‐12. The total daily dose of the probiotic mixture was 2.4 × 10^9^ CFU/mL, administered once daily. The mixture was freshly prepared, diluted with sterile water, and given by oral gavage throughout the 8‐week intervention period (Bagarolli et al. 2017). Following the first 8 weeks of the study, the vitamin D supplement was applied to the HFD + P + VD by subcutaneous injection of 0.5 mL/rat twice a week from a 3000 IU/mL vitamin D solution to achieve a concentration of 5000 IU/kg/rat. The dose was prepared fresh, based on body‐weight–adjusted calculations, to ensure consistent delivery. Vitamin D3 (cholecalciferol, active form of vitamin D) was administered subcutaneously to the experimental group. This method was chosen to ensure adequate bioavailability and to mimic clinical vitamin D supplementation practices, which may impact gut microbiota composition and obesity‐related parameters. Vitamin D3 was administered subcutaneously in its pure form without using any carrier substance (Choi et al. 2013). Serum 25‐hydroxyvitamin D levels were not measured in this study; instead, vitamin D activity was evaluated indirectly through VDR gene expression analysis.

### 2.5 Biochemical Analyses

At the end of the 16‐week feeding period, all rats were anesthetized by subcutaneous administration of xylazine (10 mg/kg; Rompun, Bayer). Under anesthesia, the rats were positioned supine and securely fixed. Subsequently, euthanasia was performed by decapitation, and maximum volumes of blood were collected immediately for biochemical analyses. Fasting plasma glucose (FPG) and insulin were analyzed as markers of diabetes. Leptin values were evaluated as a marker of obesity. High‐density lipoprotein (HDL) cholesterol, triglyceride, low‐density lipoprotein (LDL) cholesterol, and total cholesterol (TC) levels were measured to determine the lipid profile. The levels of cytokines C‐reactive protein (CRP), interleukin‐6 (IL‐6), tumor necrosis factor‐α (TNF‐α), and interleukin‐10 (IL‐10) were measured as markers of inflammation in serum samples. Insulin resistance was evaluated as follows: insulin resistance = [(fasting insulin [μU/mL]) × (fasting glucose [mmol/L]/22.5)]. Serum biochemical parameters were determined using commercially available ELISA kits according to the manufacturer's instructions.

### 2.6 Vitamin D Receptor (VDR)

In this study, VDR expression was assessed by quantitative polymerase chain reaction (qPCR) to evaluate VDR activation following vitamin D3 supplementation. This approach enabled precise measurement of VDR gene expression in target organs.

### 2.7 Collection of Fecal Samples and Microbiota Composition Analyses

Fecal samples were collected before sacrifice, placed in sterile plastic tubes using a special spoon, and frozen at −80°C. Molecular microbiological analysis of fecal samples from all rat groups was performed using quantitative real‐time PCR (qRT‐PCR) after DNA extraction, as described below. DNA was extracted from rat fecal samples using the instructions in the manual for the bottom collection tube. Targeted sequence analysis from the bacterial 16S ribosomal RNA gene was carried out using the Quick‐Quick‐16S NGS Library Preparation Kit. The bacterial 16S primers were amplified from the V3‐V4 region of the 16S rRNA gene. The sequencing library was prepared on real‐time qRT‐PCR machines, providing an innovative approach to library preparation and preventing chimeric formation. The library was constructed using the final PCR products, as measured by qPCR fluorescence reads. The final library was sequenced on Illumina MiSeq with a v3 reagent kit (600 cycles). Sequencing was performed with > 10% PhiX peak. In the analysis of stool samples, bacterial DNA specific to the given probiotic species was detected, confirming that the probiotics reached the intestinal environment alive.

### 2.8 Statistical Analyses

Quantitative data were obtained by calculating the means (X̄) and standard deviations (SD) for the study groups. The normal distribution of the groups was evaluated using the Shapiro–Wilk test. Parameters showing a normal distribution were evaluated with one‐way ANOVA (Tukey test for pairwise comparisons). In contrast, parameters that did not show normal distribution were evaluated using the Kruskal–Wallis test (Mann–Whitney *U* test for paired comparisons). The data were analyzed using SPSS 23. A value of *p* < 0.05 expressed the level of significance. The minimum sample size to be used was determined as six rats per group, based on a 95% confidence interval, using similar literature and taking into account leptin values, with a test power of 0.80, a standard deviation of 0.35, and a significance difference of 0.55 (Desmarchelier et al. 2013).

## 3 Results

### 3.1 Body Weight and BMI

Descriptive statistics for the groups' anthropometric measurements are presented in Table 1. All groups differed in weight and BMI at baseline, 8 weeks, and 16 weeks (*p* = 0.018; *p* < 0.001; *p* = 0.006). After 16 weeks, a difference was observed in pairwise comparisons between the HFD and the control (*p* = 0.004). No difference was found in the pairwise comparisons of the HFD with the HFD + P and HFD + P + VD. There was a difference in the pairwise comparisons of the HFD with the control during the first and second 8 weeks (*p* < 0.001; p < 0.001).

**TABLE 1:** Weight gain, BMI, and body weight of the groups.

	Control (*n* = 6)	HFD (*n* = 6)	HFD + P (*n* = 6)	HFD + P + VD (*n* = 6)	*p*
*Body weight (g)*					
Baseline	270.17 ± 4.66	273.17 ± 4.55^a^	271.00 ± 10.87	272.83 ± 19.10	0.018*
Week 8	289.00 ± 3.65	358.00 ± 7.47^a^	381.67 ± 14.94	413.83 ± 28.41	< 0.001*
Final (week 16)	336.67 ± 4.60	408.33 ± 10.98^a^	429.83 ± 13.28	434.50 ± 34.26	0.006*
Weight gain (first week)	18.83 ± 4.13 (6.97%)	84.83 ± 3.78 (31.05%)	110.67 ± 21.14 (40.84%)	141.00 ± 33.52 (51.68%)	0.023*
Weight gain (second 8 weeks)	47.67 ± 3.24 (16.49%)	50.33 ± 8.97 (14.06%)	48.17 ± 9.02 (12.62%)	20.67 ± 7.56 (4.99%)	0.036*
*BMI (g/cm* ^*2*^ *)*					
Baseline	0.50 ± 0.01	0.52 ± 0.01^a^	0.51 ± 0.02	0.52 ± 0.04	0.018*
Week 8	0.55 ± 0.01	0.70 ± 0.01^a^	0.72 ± 0.03	0.78 ± 0.05	< 0.001*
Final (week 16)	0.64 ± 0.01	0.77 ± 0.02^a^	0.81 ± 0.03	0.82 ± 0.06	0.006*

Abbreviations: BMI, body mass index; HFD, high‐fat diet, HFD + P, HFD with probiotic supplementation group; HFD + P + VD, HFD with probiotic and vitamin D supplementation group.

* One‐way ANOVA, *p* < 0.05 (Compared all groups).

^a^ *p* < 0.05 (Control‐HFD).

**FIGURE 2:**
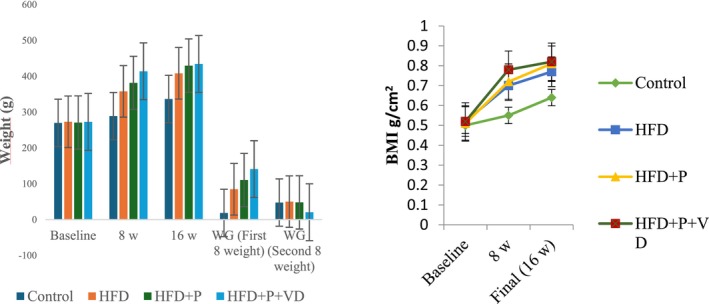
Changes in weight and BMI of the rats during the study. HFD, high‐fat diet; HFD + P, HFD with probiotic supplementation group; HFD + P + VD, HFD with probiotic and vitamin D supplementation group.

The values for weight, weight gain, and changes in BMI determined during the study are shown in Figure 2. The average weight gain percentages of the rats in the HFD, HFD + P, and HFD + P + VD during the second 8 weeks were 14.06%, 12.62%, and 4.99%, respectively. BMI increased in the HFD during the study period; however, this change was not statistically significant. In contrast, BMI changes in the HFD + P and HFD + P + VD showed a more limited increase over time. In particular, BMI and weight gain values in the HFD + P + VD were lower than those in the other groups, although these differences were not statistically significant (*p* > 0.05).

### 3.2 Evaluation of Biochemical Parameters

Descriptive statistics for biochemical parameters of the groups are presented in Table 2. There was a difference in triglyceride values between HFD + P + VD and HFD (*p* = 0.024). A difference was found between the HFD and HFD + P + VD in leptin levels (*p* = 0.016). No statistically significant differences were observed in the other parameters (*p* > 0.05). However, VDR levels showed a tendency to increase, particularly in the HFD + P.

**TABLE 2:** Evaluation of biochemical parameters of groups.

Parameters	Control	HFD	HFD + P	HFD + P + VD	*p*
FPG [mg/dL]	193.18 ± 4.73	224.53 ± 10.65	225.22 ± 13.88	213.33 ± 11.01	0.148*
Insulin [mIU/L]	21.10 ± 2.97	29.19 ± 4.82	19.41 ± 2.46	16.54 ± 3.36	0.311^e^
HOMA‐IR	18.36 ± 2.92	29.76 ± 5.63	19.95 ± 3.50	15.65 ± 3.00	0.420^e^
TC [mmol/L]	2.54 ± 0.24	3.94 ± 1.46	3.78 ± 0.97	5.64 ± 1.34	0.300*
TG [mmol/L]	2.27 ± 0.24	4.78 ± 1.73	1.80 ± 0.17	1.36 ± 0.18^c^	**0.024** ^e^
LDL‐C [μg/mL]	6.76 ± 0.52	6.03 ± 0.72	6.33 ± 0.20	6.63 ± 0.26	0.838^e^
HDL‐C [ng/mL]	63.53 ± 14.54	89.10 ± 17.21	68.64 ± 6.27	60.13 ± 13.29	0.445*
IL‐6 [pg/mL]	216.76 ± 19.46	312.22 ± 61.10	283.83 ± 81.33	223.43 ± 73.9	0.602^e^
IL‐10 [pg/mL]	397.31 ± 66.88	800.32 ± 290.56	377.59 ± 31.49	282.31 ± 45.97	0.289^e^
CRP [mg/L]	3.87 ± 0.75	6.25 ± 1.45	5.12 ± 0.44	4.43 ± 0.74	0.611^e^
TNF‐α [ng/L]	348.7 ± 148.52	572.83 ± 120.58	491.99 ± 72.99	597.71 ± 12.19	0.600*
Leptin [ng/L]	397.10 ± 60.73	717.53 ± 222.19	363.68 ± 46.41	249.82 ± 36.26^c^	**0.043** *
VDR [ng/mL]	60.10 ± 4.38	67.44 ± 7.23	78.90 ± 9.66	76.10 ± 10.10	0.593*

*Note:* Bold *p* values indicate statistically significant differences among the groups (*p* < 0.05).

^a^ *p* < 0.05 (Control‐HFD).

^b^ *p* < 0.05 (HFD‐HFD + P).

^c^ *p* < 0.05 (HFD‐HFD + P + VD).

^d^ *p* < 0.05 (HFD + P‐HFD + P + VD).

^e^ Kruskal–Wallis test, *p* < 0.05 (Compared all groups).

* One‐way ANOVA, *p* < 0.05 (Compared all groups).

### 3.3 Evaluation of Gut Microbiota

**FIGURE 3:**
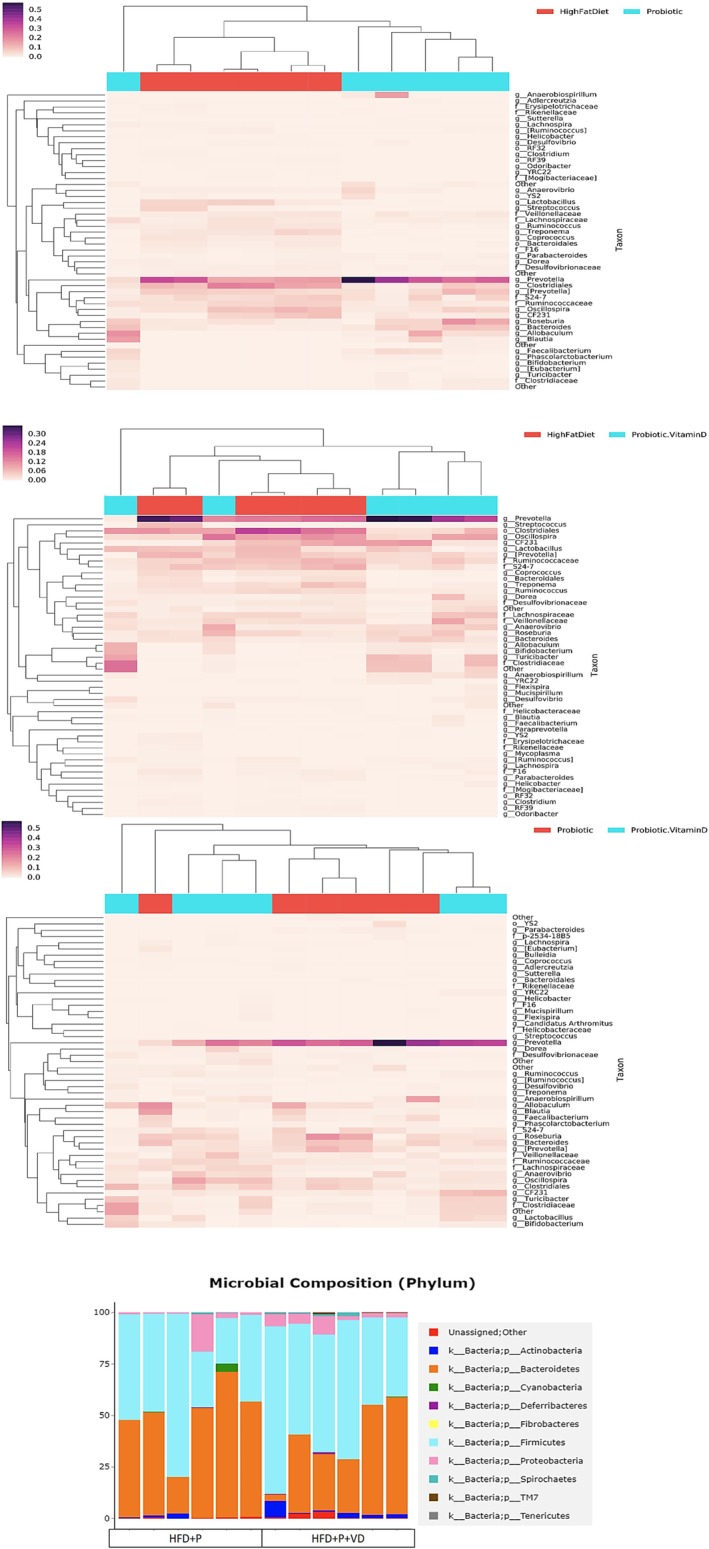
Microbiota composition in the groups after 8 weeks of dietary intervention. Rat group affiliation is indicated in orange/light red for the HFD and light blue for the HFD + P. Microbiota composition was determined in feces from the HFD + P and HFD + P + VD after 8 weeks of dietary intervention. Phylum affiliation of the genera is indicated by pink for *Proteobacteria*, light blue tones for *Firmicutes*, dark blue for *Actinobacteria*, purple for *Deferribacteres*, and orange for *Bacteroidetes*.

Compared to the HFD, a decrease in the density of *Lactobacillaceae* and *Desulfovibrionaceae* and an increase in the density of *Prevotellaceae* and *Bifidobacteriaceae* were detected in the HFD + P. Compared to the HFD, a decrease in the density of *Lactobacillaceae* and an increase in the density of *Prevotellaceae, Desulfovibrionaceae*, and *Bifidobacteriaceae* were detected in the HFD + P + VD. Compared to the HFD + P, a decrease in the density of *Prevotellaceae* and an increase in the density of *Lactobacillaceae, Desulfovibrionaceae*, and *Bifidobacteriaceae* were detected in the HFD + P + VD. Microbiota composition in HFD + P and HFD + P + VD is shown in Figure 3.

**FIGURE 4:**
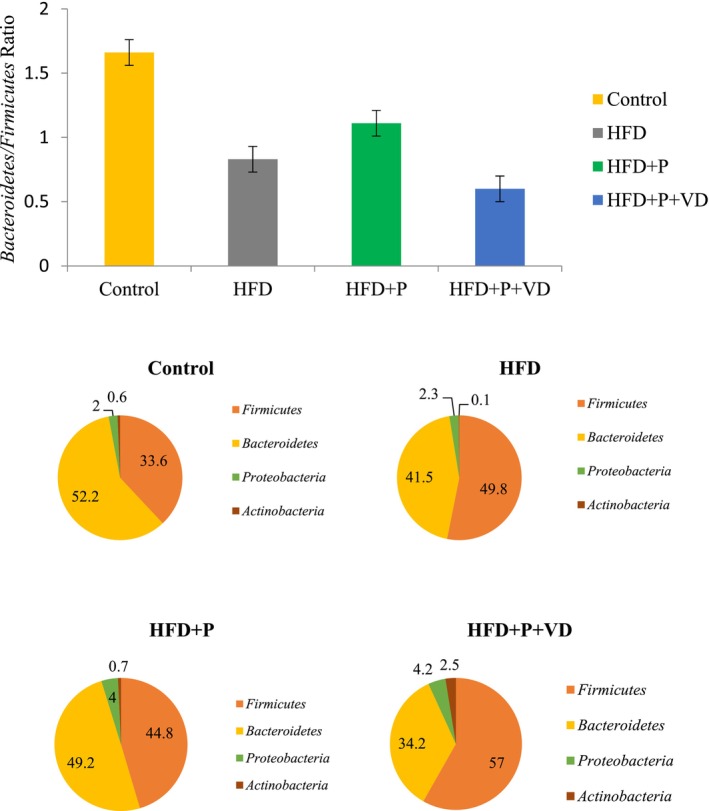
Probiotics and probiotics with vitamin D supplements altered the groups' relative levels of Bacteroidetes, Firmicutes, Proteobacteria, and Actinobacteria.

There was an increase in the phyla *Firmicutes* and *Proteobacteria* in the HFD and a decrease in the phyla *Bacteroidetes* and *Actinobacteria*, compared to the control. In addition, a reduction in the ratio of *Bacteroidetes/Firmicutes* was determined (*p* < 0.05). Compared to the HFD, a decrease in *Firmicutes* and an increase in *Proteobacteria*, *Bacteroidetes*, and *Actinobacteria* were found in obese rats given probiotic supplementation. In addition, an increase in the ratio of *Bacteroidetes/Firmicutes* was determined (*p* < 0.05). Compared to the HFD, an increase in *Firmicutes*, *Proteobacteria*, and *Actinobacteria* and a decrease in *Bacteroidetes* were detected in the HFD + P + VD. A reduction in the ratio of *Bacteroidetes/Firmicutes* was determined (*p* < 0.05). Bacterial changes in the groups are shown in Figure 4.

## 4 Discussion

Experimentally obese models are used to determine obesity and obesity‐related complications in experimental animal studies. For this purpose, high‐fat diets frequently induce obesity in experimental animal models (Bian et al. 2022; Wickramasinghe et al. 2022). In the present study, obesity was induced in the rats by applying a high‐fat diet containing 60% fat.

It has been reported that vitamin D may affect obesity and weight gain (Fraemke et al. 2022). In a 21‐week study in which obese rats were given vitamin D supplementation to their standard diet and a high‐fat diet, a significant decrease in weight was observed in the vitamin D‐supplemented group compared to the high‐fat diet group (Farhangi et al. 2017). Similarly, our results show that vitamin D supplementation with probiotics reduces weight gain. Although studies evaluating the combined effects of probiotics and vitamin D remain limited, available evidence indicates heterogeneous outcomes across related interventions. In this context, a recent meta‐analysis of randomized controlled trials reported that probiotic supplementation significantly reduced body weight, waist circumference, and visceral fat, while showing no significant effect on BMI or LDL‐C levels, suggesting more pronounced effects on central adiposity than on overall BMI. Nevertheless, considerable heterogeneity among the included studies was emphasized (Guo et al. 2025).

A significant decrease in FPG and HOMA‐IR levels was reported in the vitamin D‐supplemented group compared with the control groups in mice with obesity induced by a high‐fat, high‐sugar diet (Benetti et al. 2018). Vitamin D supplementation with probiotics is thought to improve markers of diabetes. Studies show that dyslipidemia, among the complications of obesity, is associated with vitamin D (Zhang et al. 2022). In a study examining the effects of vitamin D on obese mice, decreases in total cholesterol, triglycerides, and LDL levels were observed after vitamin D supplementation, with a significant difference observed only in LDL levels (Benetti et al. 2018). Vitamin D affects inflammation through various mechanisms (Giménez et al. 2022; Śledzińska et al. 2022). In a study in which obese mice were fed a high‐fat and high‐sugar diet for 4 months, vitamin D supplementation reduced TNF‐α levels (Benetti et al. 2018). According to the data of our study, total cholesterol and LDL levels were higher, and triglyceride and HDL levels were lower in the HFD + P and HFD + P + VD. Moreover, IL‐6, IL‐10, and CRP levels were lower, and TNF‐α levels were higher in the HFD + P + VD. We found a significant difference in triglyceride levels compared with the HFD. TNF‐α and lipid levels other than triglycerides may have been influenced by the continued high‐fat diet during the intervention period, potentially attenuating the potential consequences of taking vitamin D and probiotics on these parameters. Therefore, vitamin D may have limited effects on lipid and inflammatory profiles under the present experimental conditions. Furthermore, in our study, probiotic and vitamin D supplementation decreased leptin resistance by significantly lowering leptin levels, a marker of obesity. Additionally, the serum VDR levels were higher in the HFD + P + VD compared to the HFD. The serum VDR levels in the HFD + P + VD were lower than those in the HFD + P. This finding may be explained by potential interactions between probiotic supplementation and vitamin D–related signaling pathways, including VDR modulation. In addition to these results, the literature reports conflicting findings regarding vitamin D's impacts on obesity and metabolic processes, and evidence remains inconclusive. Vitamin D supplementation often has minimal impact on metabolic and anthropometric outcomes in individuals with obesity and related metabolic diseases, according to a recent comprehensive review and meta‐analysis of randomized controlled trials. There were no notable alterations found in most parameters, including lipid profile, glycemic indicators, waist circumference, and body weight, although limited effects were noted in some subgroup analyses (Ge et al. 2025). A randomized controlled trial investigating combined supplementing with probiotics and vitamin D in women with polycystic ovarian syndrome reported improvements in insulin sensitivity and inflammatory markers; however, no significant changes were observed in fasting glucose or other key metabolic parameters such as AMH, suggesting that the effects of combined supplementation may be parameter‐specific rather than globally effective (Banikazemi et al. 2025). A systematic review of seven randomized controlled trials based on PRISMA suggests that combined supplementation with vitamin D and probiotics may deliver greater overall health benefits compared to either intervention alone, including improvements in metabolic, inflammatory, and immune system outcomes; however, it emphasizes the need for further studies to validate optimal formulations and clinical applications (Abboud et al. 2020).

It has been reported that the intestinal microbiota changes in response to a high‐fat diet (Hassan et al. 2022; Yang et al. 2022). In another study, the density of *Firmicutes* increased in the high‐fat‐fed group compared with the low‐fat‐fed group, and the density of *Bacteroidetes* decreased (Kusumoto et al. 2017). Our results are consistent with the general literature. Compared to the control, the HFD showed a significant increase in Firmicutes and Proteobacteria, while a substantial decrease was observed in *Bacteroidetes* and *Actinobacteria*. In addition, the ratio of Bacteroidetes to Firmicutes was reduced, and the dominant bacterial species across all groups were *Firmicutes, Bacteroidetes*, and *Proteobacteria*. However, it should be noted that the *Bacteroidetes/Firmicutes* ratio was considered as part of overall gut microbiota composition changes rather than as a standalone indicator.

Probiotic supplementation in a high‐fat diet alters the intestinal microbiota (Lof et al. 2022; Zafar et al. 2022). In a previous study, compared to a standard diet and a high‐fat diet, probiotic supplementation increased the density of Firmicutes and Actinobacteria. It decreased *Bacteroidetes* density compared to the control groups (Bagarolli et al. 2017). In another study, probiotic supplementation containing *Lactobacillus* and *Bifidobacterium* strains to obese rats increased the density of *Bacteroidetes*, *Lactobacillus*, and *Bifidobacterium* while reducing the density of *Firmicutes* (Shin et al. 2018). As in the last study, in our study, compared to the HFD, significant decreases in Firmicutes and significant increases in Proteobacteria, Bacteroidetes, and Actinobacteria were observed in the HFD + P. In addition, substantial increases in *Firmicutes*, *Proteobacteria*, and *Actinobacteria* and a significant decrease in *Bacteroidetes* were detected in the HFD + P + VD compared to the HFD. Our results may contribute to the existing body of knowledge in several aspects. First, probiotic supplementation may influence intestinal microbiota composition in the context of diet‐induced obesity. Second, probiotic and vitamin D co‐supplementation were associated with changes in intestinal microbiota diversity in obese rats. It has positive effects, particularly on the amounts of Actinobacteria and Bifidobacteria, which are known as beneficial bacteria for health. Third, probiotic supplementation with vitamin D tends to reduce leptin resistance, a marker of obesity. Finally, one of the most important findings of this study is that probiotic supplementation may enhance the biological effects of vitamin D, potentially by modulating VDR expression and related pathways. Consistent with this hypothesis, a randomized controlled clinical trial in patients with irritable bowel syndrome without constipation reported that combined probiotic and vitamin D supplementation improved gut barrier integrity and modulated microbiota composition, accompanied by increased beneficial bacterial populations and reduced intestinal permeability markers, supporting a potential synergistic effect of these interventions on gut‐related functions (Laterza et al. 2025).

This study has some limitations that should be considered when interpreting the findings. This study has some limitations. A limitation of this study is the relatively small sample size (*n* = 6 per group), which may reduce statistical power and limit the generalizability of the findings. This may also have constrained the ability to detect subtle differences in biochemical and gut microbiota parameters between groups. In addition, serum 25‐hydroxyvitamin D levels were not measured, so systemic vitamin D status could not be directly correlated with VDR expression. The study was conducted in an experimental animal model with a relatively controlled design, which supports internal validity but may limit direct extrapolation to humans. Furthermore, the continuation of a high‐fat diet during intervention period may have attenuated or masked the potential impact of vitamin D and probiotic supplements on inflammatory and metabolic consequences. Although relevant metabolic and molecular parameters were evaluated, the dosing strategy (single regimen and fixed administration protocol) may also limit interpretation of dose–response relationships. Although relevant metabolic and molecular parameters were evaluated, the mechanisms underlying the interaction between probiotics and vitamin D require further investigation.

## 5 Conclusion

In conclusion, this study provides experimental evidence that probiotic and vitamin D supplementation may influence metabolic parameters and gut microbiota composition in diet‐induced obesity. The observed changes in VDR levels and microbiota profiles suggest a potential interaction between these interventions. From a translational perspective, these findings may support further investigation of combined supplementation strategies as a complementary approach to obesity‐related metabolic disturbances; however, any potential application should be considered cautiously given the study's experimental nature. Further well‐designed studies are needed to clarify the underlying mechanisms and potential clinical relevance of these effects.

## Author Contributions

**Mehtap Ünlü Söğüt:** conceptualization, investigation, funding acquisition, methodology, validation, visualization, writing – review and editing, project administration, supervision, data curation, resources. **Gül Eda Kılınç:** conceptualization, investigation, funding acquisition, writing – original draft, methodology, validation, visualization, writing – review and editing, software, formal analysis, project administration, data curation, resources.

## Funding

This study was supported by the Ondokuz Mayıs University Scientific Research Projects Coordination Unit (OMU, BAP, Project Number: PYO.SBF.1904.17.007).

## Disclosure

Author approval and responsibility statement: All authors have read and approved the final version of the manuscript. The corresponding author had full access to all data in this study and takes full responsibility for the integrity and accuracy of the data analysis.

## Ethics Statement

The ethical approval for this research was obtained from the University Ethics Commission (No: 68489742–604.01‐E.29664).

## Consent

The authors have nothing to report.

## Conflicts of Interest

The authors declare no conflicts of interest.

## Data Availability

The data that support the findings of this study are available on request from the corresponding author. The data are not publicly available due to privacy or ethical restrictions.
